# Why do older people not use the public health services of the integrated aging program? A multidimensional approach in a qualitative study

**DOI:** 10.1186/s12913-022-08689-6

**Published:** 2022-10-25

**Authors:** Amir Mohamad Moghadasi, Shima Sum, Hossein Matlabi

**Affiliations:** 1 Department of Geriatric Health, Faculty of Health Sciences, Tabriz University of Medical Sciences, Attare Neishabouri St, Tabriz, 5165665811 Iran; 2 Department of Public Health, School of Public Health, Babol University of Medical Sciences, Babol, Iran; 3 Research Centre for Integrative Medicine in Aging, Aging Research Institute, Tabriz University of Medical Sciences, Tabriz, Iran

**Keywords:** Older people, Health care services, Iran, Qualitative study, Integrated aging program

## Abstract

**Objectives:**

The world’s population is aging rapidly and a huge amount of services are being provided to meet the needs of the older people. Identifying the factors affecting the non-attendance of the older people to health care centres is of particular importance. We focused on the reasons why older people do not use the services of the integrated aging program in Iran from the perspective of the older people, general practitioners, and primary health providers.

**Methods:**

A qualitative study in Ghaemshahr (IRAN) carried out during 2021. Data were collected through semi-structured interviews in two groups with the participation of 29 older adults and 18 employees of the health centres Purposeful sampling and sample size were determined based on data saturation. Data were analyzed manually using conventional content analysis.

**Results:**

Potential barriers to and challenges of older adults were generally categorized into four main themes including individual, systemic-structural, environmental, and social factors.

**Conclusions:**

Both groups agreed on many aspects, including lack of education of the patients and lack of proper medical services. Existing problems in health care relate to both medical and non-medical factors. Improvement in health care delivery requires a deliberate focus on the patients’ specific needs.

**Supplementary Information:**

The online version contains supplementary material available at 10.1186/s12913-022-08689-6.

## Introduction

The United Nations gives prominence to the older people's adequate access to health services to help them maintain or restore an optimal level of physical well-being [1]. The health care system must be prepared to respond effectively to the problems caused among older adults. However, the Ministry of Health and Medical Education (Iran) cannot meet the needs of the older people alone, and insurance organisations should cooperate [2]. An integrated geriatric care program is currently being implemented in the country's health centres. The program offers outpatient treatment which usually focus on one health area and provide expert preventative care and diagnoses [3]. According to studies, the Iranian health network's inability to meet clients' needs and the lack of public referral and acceptance of urban health centres are critical challenges [4]. In Iran, in recent years, despite the great attention to the development of comprehensive health care centres in cities, unfortunately, the number of people referring to the centres has decreased in some cases [5].

Benefiting from health services as the intersection of the supply and demand side of healthcare and well-being has been an important issue in health policies [6]. Access to health care is considered as a fundamental right and a social goal. This means all people are entitled to health care, even if they do not need it [7]. A person's quality of life in old age depends mainly on the access to and use of health services, employment and income, social support, and educational opportunities [8, 9]. Accessibility is a substantial component of the quality of primary health care services and is often conceptualised in terms of the availability, appropriateness, acceptability, and cost-effectiveness of care [10–12]. Due to the high workload and lack of trained staff in many centres, there is a passive approach toward healthy and independent older people [13]. Access to public health services may be challenging for the older people due to deteriorating health, reduced physical and social mobility, and limited financial ability [14]. Previous studies have shown that referrals to health centres in cities are less than in villages [15, 16]. Lack of awareness about available services is one of the leading and intervening causes [17]. Low health literacy is associated with patients who are older, have limited education, lower income, chronic conditions and those who are non-native Persian speakers. Low health literacy covers a wide range of perceived causes of not referring to the health centres, including lack of understanding of health problems, prevention approaches, and the importance of follow-up [17]. There is a general perception that patients only come to the clinic with acute symptoms. Moreover, only a small number of people volunteered to use health promotion or prevention activities [18]. The physical environment of many health centres is not suitable and does not create a good feeling in the patients [19].

It has been found that the use of health services in low-income countries is influenced by health insurance, having a chronic illness, age, gender, educational status, and living in the city [20–22], distance from the health care centre, availability, reasonable costs, and quality of health care [23]. The lack of proper equipment and physical structure is one of the deterrents to providing the older people with health care. A suitable physical space may play an influential role in improving the quality of services by creating a positive outlook for recipients and service providers [24]. Furthermore, a software bug in the service delivery system causes both congestions in the centres and customers' dissatisfaction [17]. International studies revealed that movement disorders, paraclinical procedures costs, distrust in general practitioners, and neglect of older adults were the main barriers to healthcare access [17, 25, 26].

As mentioned above, regardless of their age, older people with chronic conditions often face multiple and complex challenges when trying to use the routine health facility. Therefore, we aim to gain a better understanding of the barriers faced by older people and explain*’why do older people not refer to public health services centres?’ ‘why do older people not use the services of the national integrated aging program?’ ‘what are the barriers and challenges?’*.

## Methods

### Study design

A descriptive qualitative study was conducted in Ghaemshahr, Iran during 2021.

### Participant recruitment

Potential and eligible participants (29 older adults and 18 employees of the health centres) were selected by purposive sampling. We tried to recruit the people who had the most suitable information about the study with maximum diversity which includes the targeted selection of cases with a wide range of differences in the desired aspects. The respondents were consisted of two groups including older people and healthcare workers (general practitioners and primary care providers). Negotiations were held with the directors of the health centres to provide all the facilities to use their scheme for fieldwork. By visiting the centres and using the integrated health system, the profile of all older adults was investigated to recruit potential participants. The inclusion criteria of the older people group included the age of 60 and over, having a registration number in the health centre, consent to participate in the study, and at least six months of residence in Ghaemshahr. They were ineligible if they had a history of cognitive and psychological problems like major depression, dementia, bipolar disorder, and schizophrenia which diagnosed by an urban general practitioner and referred to the centre's clinical psychologist. Furthermore, among the general practitioners and primary care providers, employees who had experience working with the older adults and had desire to express their opinions and views toward the subject of research were invited to contribute in the study. In the older people group, 45 participants were approached, two of whom withdrew from the study during the interview, and fourteen older people refused to be interviewed. Finally, the data were collected from 29 older adults. In the group of general practitioner and health care providers, the opinions of 18 participants were obtained.

### Procedures and data collection

The data was collected using in-depth and semi-structured interviews which were administered face-to face. Each interview lasted on average for 30- 45 min were carried out with the participants in a private area. Every meeting began with introducing the research team, explaining the study's purpose, and thanking the participants for their presence in the interview despite the conditions caused by COVID-19 pandemic. Then, sociodemographic and the key questions in line with the purpose of the research were asked such as ‘*why older people do not use the services of the integrated aging program*?’ ‘*what do you think about the changes and barriers*?’. Furthermore, follow-up and exploratory questions including ‘*what are the motivational factors influential in welcoming the program*?’ ‘*why does not the older people go to the centres?*’ ‘recommendations for overcoming or reducing these barriers?’. During the interview sessions, participants were encouraged with phrases such as "please explain more, give examples" and so on. With the written informed consent of the participants, all interviews were audio-recorded, subsequently transcribed and notes were taken on the non-verbal cues. Data saturation was operationalized in a way that was consistent with the research questions (no additional issues or insights were identified).

### Thematic analysis

The data was analyzed manually using the technique that proposed by Braun and Clarke in 2006 [27]. The six steps of this method included familiarizing the researcher with the data, generating initial codes from the data (generating initial codes), searching for themes by reviewing the various codes extracted in the previous steps, reviewing the themes and re-comparing them with the data to ensure accuracy (reviewing themes), defining and naming the main themes, and preparing for the final report [27, 28].

### Data trustworthiness

Credibility, transferability, dependability, and confirmability were applied to validate the data. Specific strategies were used to attain trustworthiness such as long contact with the research environment, continuous observation, inspection from different angles, exchange of views with peers, analysis of negative cases, adequacy of references, control by members as well as characteristics of the investigator and the abilities for clarification, summarization, development of the data set during the collection phase, and use of special coding procedures [29].

### Ethical considerations

The present study was checked and approved by the Ethics and Research Committee of Tabriz University of Medical Sciences (IR.TBZMED.REC.1400.068). Witten informed consent was individually obtained, dated, and signed from all the participants. The purpose of the interviews and the reason for using the tape recorder were clearly explained. The participants were also assured that they could leave the research at any point. Finally, the research team attempted to maintain the participants' identities and other information with complete confidentiality.

## Results

The sociodemographic characteristics of the respondents are presented in Tables 1  and 2. The group of older people was 29 persons and not so old (60–64 years old). The participants consisted of 19 men (65.5%) and ten women. A total of 72.4% of the respondents have three or four children and predominantly live with their spouse and family members. The largest number of older adults (82.8%) were married and financially independent. Regarding educational level, approximately two-thirds of the participants were ranged from secondary to tertiary stages. Finally, all respondents were supported by one of the main social security, state or armed forces funds and medical services insurance.

**Table 1:** Socio- demographic characteristics of the older people (*n* = 29)

Variables	Frequency	Per cent(%)
**Gender**		
Male	19	65.5
Female	10	34.5
**Age groups**		
60–64 years old	17	58.7
65–69 years old	9	31
70–74 years old	0	0
75–79 years old	2	6.9
≤ 80 years old	1	3.4
**Level of education**		
Illiterate	2	6.9
Primary/ Secondary	7	24.1
College	11	37.9
Academic	9	31
**Number of children**		
1 -2	3	10.3
3—4	21	72.4
5—6	4	13.8
≤ 7	0	0
N/A	1	3.4
**Marital status**		
Single	0	0
Married	24	82.8
Divorced	2	6.9
Widowed	3	10.3
**Living arrangement**		
Spouse	9	31
Children	2	6.9
Family members	15	51.7
Relatives	2	6.9
Others	1	3.4
**Occupation**		
Housekeeper	5	17.3
Self-employed	8	27.5
Retired	16	55.2
**Financial dependence on family members**		
Yes	5	17.2
No	24	82.8
**Insurance status**		
Social security	12	41.4
State	14	48.3
Armed forces	3	10.3

**Table 2:** Demographic characteristics of physicians and healthcare providers (*n* = 18)

Row	Gender	Position	Age	Duration of employment	Academic degree	Duration of activity in the center
1	Female	PHP^a^	49	15	Bachelor of Midwifery	8
2	Female	PHP	36	13	Master of Health Services Management	10
3	Female	GP	29	3	Medical doctor	2
4	Female	GP	47	20	Medical doctor	11
5	Female	PHP	32	10	Bachelor of Midwifery	8
6	Male	GP	52	22	Medical doctor	6
7	Female	PHP	22	6 Month	Bachelor of Public Health	6 Month
8	Female	PHP	37	14	Bachelor of Nursing	4
9	Female	GP	47	21	Family physician specialist	2
10	Female	PHP	47	25	Midwifery Associate	7
11	Female	PHP	39	15	Bachelor of Nursing	9
12	Female	GP	38	11	Medical doctor	2
13	Female	GP	45	16	Family physician specialist	2
14	Female	GP	47	21	Medical doctor	15
15	Female	PHP	44	20	Bachelor of Nursing	12
16	Female	PHP	48	16	Bachelor of Midwifery	1
17	Female	PHP	45	20	Bachelor of Public Health	6
18	Female	GP	55	26	PhD in Reproductive Health	6

^a^ Primary healthcare provider

Furthermore, in the group of healthcare workers eight general practitioners and ten primary health providers were interviewed face to face and individually. The majority of participants (*n* = 17; 94.4%) were women.

Potential barriers to and challenges of older adults were generally categorized into four main themes including individual, systemic-structural, environmental, and social factors. In the following, the main and sub-themes are described and documented as much as possible based on the participants’ statements (see Additional file 1).

### Viewpoints of older people

#### Individual factors

This category comprises specific aspects including following up only in the case of getting sick, self-medication, the idea of services uselessness, the preference or need to see a specialist, lack of knowledge about services, physical weakness, and having general practitioners in the family.

Lack of knowledge about the disease progression and outcomes might intensify the disease. One of the participants stated:

*“…Usually, if I get sick, I refer to a centre. It is not like they ask me to refer, for example, every three months. They do not call, so I do not refer them unless needed. They do not care about the patient, whether you are okay or are not...”*.

Regarding self-medication, one participant said:

*“…For example, sometimes I have a heartbeat. Since my wife suffers from it, I take the same pills that they give her…”.*

The uselessness of services is repeatedly mentioned by the older adults. One of the participants said:

*“…They do nothing, but I go to get a prescription and take medication…”.*

Evaluation of older adults usually differs from a standard medical evaluation. For older patients, especially those who are very old or frail, history-taking and specialized examination may have to be done at different times. Generally, most of people believe that the knowledge and skills of specialist physicians are greater than those of general practitioners. Regarding the preference or necessity of referring to a specialist, one of the respondents clarified:

*“…When a specialist takes $2 for 5 min, the quality of her/his services is different. If s/he takes 15 cents, all of us will go inside…”.*

Another participant said:

“… *I'm sick, and I have to go to my doctor. I cannot see any general practitioner, so I go to a specialist more often...”*.

Most of the older people were not aware of the services available by the centres. One of them said:

*“…We do not have diabetes or other diseases; we do not deal much with family doctors…”.*

A further participant talked about having a physician in the family:

*“…My children are doctors. I do not need to refer to the centres. My daughter and brother are physicians, and his wife and nephew are pharmacists. If I need to see a doctor, I will refer a specialist. I do not need to go to a general practitioner...”.*

#### Systemic-structural factors

This category contains a number of sub-themes, including lack of informing and calling for services, referral to other organizations or another therapist, absence of general practitioners, restrictions on prescribing, administering or supplying drugs, limited availability and affordability of mental health care services, lack of nutrition counselling and injection services, low-quality equipment, E-prescribing errors, lack of necessary doctor's expertise, the ineffectiveness of medical insurance, the small effect of doctor's prescription on the cost of drugs and tests, financial incapacity, and inefficiency of the referral system.

Most of the participants expressed their dissatisfaction with the lack of informing and calling for services provided by the centres. Two participants believed:

*“…We referred and registered for the family doctor, but I don't even know his/her name, or where s/he is. I have never visited him/her…”.*

*“…The family doctor messaged but did not call. They did nothing. Once, they messaged and asked if I was satisfied with the services or not. What should I say? I haven't referred, and you haven't done anything...”*.

Government agencies grant benefits of services and medicine to the covered individuals. One of the participants stated:

*“…We are from the armed force. The organization that provides medical services to us do not know our information. We just go to the centre and ask for registration…”.*

The absence of a doctor is unpleasant for everyone. The issue is more critical for the older people due to their physical and mental status. One of the participants said:

*“…I referred several times, but the family doctor wasn't there. One of the doctors was my neighbour. I do not name him because he was also my mother's relative. They said the doctor was not here at the time, so go and come back two hours later. A family doctor is nonsense! I go to see if s/he is there or not! That is why I don't refer to the centre anymore...”*.

Regarding the restrictions on prescribing, administering or supplying drugs, one of the participants explained:

*“…To tell the truth, I do not see the family doctor unless to renew the medicine. S/he can't do much. S/he is allowed to write only a few medicines. If I get ill, I don't refer to a family doctor…”.*

Moreover, limited availability and affordability of mental health care services, lack of nutrition counselling and injection services was frequently mentioned. It will be easier for the patients to have comprehensive health care in one place as one of the participants defined:

*“…It will be good if a nutritionist and a psychologist are there. There is no psychologist to talk with if someone has a problem or the centre lacks nutrition services…”.*

Other participants pointed to the low quality of the equipment and asked for new equipment in the centres:

*“…Their equipment is not first class. For example, the blood pressure device is not accurate. The care provider begins to test blood pressure and opens/closes it six times…”.*

Electronic prescription is a new project and causes many complaints. Due to the slow speed of the Internet and its time-consuming process, some participants have to purchase medications without a health insurance card. A participant stated:

*“…It's a big problem. We used to get medicine with a health insurance card quickly. Now they say we don't have access to the Internet; there is connection trouble, the prescription isn't readable, go back to the doctor and ask for what s/has written...”.*

Furthermore, one of the participants emphasized the dissatisfaction with the doctor in diagnosing the disease and claimed:

*“…We have to say I feel hurt here or there, s/he prescribes acetaminophen, the diagnosis is not good. Or, I must say my blood sugar is high, then s/he tells me to take this or that medicine...”.*

The ineffectiveness of medical insurance, the small effect of doctor's prescription on the cost of medications and tests costs are severe matters for clients of all ages, especially among older patients, who are usually retired and expect to be covered by their insurance and pension funds. Unfortunately, the poor performance of insurers has an adverse impact on the effectiveness of physicians' prescriptions and medical deductions.

*“…Testing is expensive. The insurer doesn't pay for the costs even with the stamp of a family doctor. The health insurance card is useless and now they do not accept the insurance card at all…”*.

*“…The health insurance card is something nonsense and useless. It is written, but we have to buy and pay which come out of our pockets. The insurer only gets the cash. It is in the interest of the insurance firm...”*.

Regarding the financial incapacity, the participants stated:

*“…Due to busyness, sometimes I have to refer to the centre or the doctor later. The cost of medications is high, so we do not go much…”.*

Finally, inefficiency of the referral system is a matter of liability for insurance companies. Many specialists do not have insurance contracts, and patients are forced to pay the costs which come out of their pockets.

*“…I referred to the specialist for my Lumbar Herniated Disc. He did not register in the system at all, so I didn't have a code to get medicine. He said because I do not have a contract with insurance, I do not write anything in the prescription. We have to pay for everything, so the insurance is useless...”*.

#### Environmental factors

The following sub-themes were identified for environmental factors: lack of space for cars, lack of health and public facilities, and unfavourable environment of the health centres.

Older people deserve health care that's easy to navigate. Hassle-free access is severe concerns for the older adults and a factor for their desire to refer to centres. A participant said:

*“…The family doctor is in the city centre. There is no parking space. We usually have to walk…”.*

The lack of public facilities and age-friendly environments are pervasive problems that affect care providers and recipients. An older man said:

*“…There is no toilet. It is only for doctors, not for patients. You cannot go there when you need it…”.*

*“…The family doctor has a small office with rotten stairs and a steep slope because the building is old…”.*

#### Social factors

This category contains specific social aspects including fear of COVID-19 pandemic, lack of social support, and inability to establish interpersonal communication by health workers.

Regarding the COVID-19 pandemic and its impact on the integrated aging program, one of the participants said:

*“…Since the coronavirus has come, I haven't referred to the health centre or doctor…”.*

About the lack of social support, one of the participants also mentioned:

*“…These days, children do not feel like their parents. I do not leave anything to children in any way...*”.

Moreover, regarding interpersonal communication skills, one of the participants stated:

*“…If you visit specialists in the office, they are concerned about all the details because they get the money and you have to come over again, but in the public centres, they behave you like an animal. They don't explain much… “.*

Another respondent explained:

*“…The previous family doctor kept me waiting. He did not allow me to speak at all. He procrastinated me. He was impatient. He just fought. I finally changed him, and I was relieved…”*.

### Viewpoints of general practitioners and primary healthcare providers

#### Individual factors

This category contains a number of sub-themes including ‘older people not taking the disease seriously’, preference or necessity of referring to a specialist, lack of knowledge about services, self-medication, physical and mental difficulties, forgetting the appointment, having a doctor in the family, and the idea of services uselessness.

Some older patients may refuse treatment because they do not understand what it involves or how it will improve their health. One of the staff said:

*“…The elderly does not take it very seriously. This needs to be addressed. They refer again only, for example, to get insulin…”.*

Concerning the preference or necessity of referring to a specialist, a doctor said:

*“…Unfortunately, some of the elderly do not believe in seeing a general practitioner at all, and they like to just refer to a specialist…”.*

A primary health provider also mentioned:

*“…For heart disease, everyone refers to a specialist. Some people call and say we just refer to our doctor…”.*

Moreover, regarding physical and mental disease, one participant said:

*“…Some people do not come because of physical incapacity. They rest just in bed and don't have the physical ability to refer to the centre…”.*

*“…Since I've retired and no longer have a position in other offices, my relatives have left me alone. I'm shocked and depressed. One of the cases said that if I were not afraid of God, I would have committed suicide...”*.

About forgetting the appointments, one of the care providers stated:

“…*Besides the pandemic that has reduced referrals, they also have memory impairment and so forget appointments. We call them as much as we can, though some are missed…*”.

Regarding having a doctor in the family members, a staff stated:

*“…There are some elderlies who no longer need to refer to the centre because they have a doctor in the family. For example, there was one case whose son was a psychiatrist doing all the medical work for his parents. I called the elderly man, and he said, “I don’t need to refer to the centre…”*.

One of the care providers said that the elderly think the services are useless:

*“…Some elderlies say you do nothing there and I don't come! Their children believe the same and say we spend money on our elderly or their care. When we are far away, we have to hire a nurse. What can you do for us? Those with such a perspective cannot meet their needs with the centre, so they prefer not to refer…”*.

#### Systemic-structural factors

Sub-themes of systemic-structural factors were identified as lack of awareness, referring to private clinics, lack of rehabilitation services, no medicine provision, no follow-ups, inadequate equipment of centres, low-quality services due to lack of staff, low speed and flawed information registration system, shortcomings of electronic prescription and disruption of medicine reception, the COVID-19 pandemic, lack of expertise in the field of aging, the inefficiency of the referral system, inefficiency of insurance, and high costs of medical procedures.

Regarding the lack of awareness, one of the doctors said:

*“…Many of older adults do not know who their family doctor is, where he works, how they can access the doctor, and what services the centre provides. They think we just provide vaccination and renew the medications. This is a drawback of the system...”*.

About referring to private clinics, a doctor stated that *“…There are so many clinics and specialists in the city that I've not seen them at all…”*. Another respondent also commented:

*“…Those with the insurance card of the Social Security Organization refer to the hospitals of the organization to reduce the costs…”*. Furthermore, regarding the lack of rehabilitation services, a health provider stated that *“…Much can be done in a public environment to encourage the elderly; for example, physiotherapy...”*.

*“…There are no special follow up programs. We have pressure control, height and weight check, but without medicine…” (A primary healthcare provider).*

A doctor who has served in a village, in comparing the service provided in the city with the village, indicated that the follow-ups in the city are deactivated:

*“…In the city, the referral is optional, but care providers go to homes and follow up in villages. We do not have this possibility in the city…”.*

About the inadequate equipment of the centres, two participants believed that *“…We don't have a glucometer kit to check the patient's sugar, so we have to ask for or send a test. Those elderly who does not have the device can’t have cared for…”*.

*“…Only blood pressure is being screened here. We do not have a device for diabetes. Centres in the village have a glucometer, but those in cities don't. The rest of the screenings are done by phone, for example, colon cancer or fit testing… “.*

The decline in the quality of services due to the lack of staff was stated in an interview as.

*“.. There is a lack of workforce in many centres. An expert may do three or four tasks for the elderly, middle-aged patients, pregnant women, and children. His/her workload is high, so the quality gets lower…”. Most physicians and care providers have expressed dissatisfaction with the low speed and defects of the information registration system. A participant stated:*

*“…The Internet is terrible. It gets disconnected repeatedly. I record on the system, and then I notice no connection. I have to do it over. This makes me tired, takes up all my time, and makes the patient wait behind the line...”*

A health worker noted the disadvantages of the E-prescription and the disruption of medications as *“…E-prescription gives error. We have to do it over all the time…”*.

Regarding to the pandemic, a participant said *“…Those who need care don't refer to the centre due to the mass vaccination…”*.

Setting a daily capacity limit may cause a lot of terrible for older adults as a health provider stated that *“…The capacity of the centres should be increased so that the elderly does not have to be covered by centres away from their home…”*.

The majority of health workers have poor knowledge on basic knowledge for physical, psychological and social changing of older people.

*“…They should hold training courses for caring for the elderly. If they don't want to hire a person with the required expertise, at least they can provide the staff with training on treating the elderly...”*.

Moreover, the system of health insurance is wasteful and inefficient. One of the doctors emphasized that “…*About 50% of the prescriptions referred to a specialist are rejected. For example, they say the insurance does not pay them or does not pay on time. This makes the elderly complain and make them dissatisfied with us…”*.

Without insurance, the cost of going to a doctor typically ranges from $30 to $60 but prices may vary depending on several factors such as lab tests, where you seek care, and procedures done at the visit. A general practitioner stated that *“…The medical costs and testing are so high that we can't afford. So, people don't refer to laboratories…”*.

Referral faults are one of the greatest issues caused by inefficient referral management systems. One of the doctors said as *“…Higher ranked doctors often do not have a contract. We had a contract with an ophthalmologist, but he cancelled. The patient refers to the centre, and we don't have the doctor…!”*.

#### Environmental factors

The following sub-themes were identified for environmental factors: lack of space for cars, lack of health and public facilities, and unfavourable environment of the health centres.

Regarding the lack of parking space for patients, one of the participants said:

*“…Our centre is located in the worst spot. There is no place to park. Because most of older people are disabled, they have to park; the person who comes with old clients has to park the car and take the old patient to the centre. Nevertheless, often there is no parking space. It's a crowded place. If the elderly can, they will come themselves, but they need family support. So, the family may not accompany them, and the number of visits gets lower...”*.

A primary health provider noted the lack of public health facilities as *“…Unfortunately, we do not have a toilet for the client in the centre. It will be much better if the centre gets facilitated with toilet…”*.

Moreover, a general practitioner stated that *“…There is only one toilet usually used by the staff. The elderly may use it only in emergencies. No water cooler, no heating and cooling device. Though, since the corridor is small, I leave the door of my room open to cool down there…”.*

Physical quality and service environment play an important role in promoting health and well-being for patients and providing supportive workplaces for staff.

*“…Two doctors are in the same room. The patients complain about the lack of privacy. Why two doctors in the same room? They even vaccinate here. It's noisy...”.* And *“…Since some cases come from a long distance, our centre is not convenient for them. There is no taxi road, and they have to walk a bit. It is difficult to get to the centre…”.*

#### Social factors

Studies have indicated that there is a fear of COVID-19 pandemic in various patient groups, healthcare workers, and the general population.

*“…Some of the cases do not refer, some refer rarely. They say we bought the medications. I ask why? They say you do corona test here. We are afraid…” (A general practitioner). Furthermore, social and cultural domains can limit the utilization of health services.*

*“…There are cases that I call their children and s/he says you do not call me anymore, it does not matter to me, s/he stays with someone else, why don't you call him/his and I asked for the number of that person, but the child says you do not call me anymore. S/he even does not give the number of the person who cares the parent…” (A primary health provider).*

Public health centres provide low cost care, are generally overcrowded, and largely used by the poor and frail older adults. People with low-incomes use fewer preventive care services.

*“…There is a travel fee. They have to come by taxi. That is expensive, so they do not come and wait for their child or someone else with a car to bring them to the centre…” (A primary health provider).*

## Discussion

We focused on the reasons why older people do not use the services of the integrated aging program in Iran from the perspective of the older people, general practitioners, and primary health providers. A qualitative study in Ghaemshahr (IRAN) carried out during 2021. Data were collected through semi-structured interviews in two groups with the participation of 29 older adults and 18 employees of the health centres. Purposeful sampling and sample size were determined based on data saturation. Data were analyzed manually using conventional content analysis. Potential barriers to and challenges of older adults were generally categorized into four main themes including individual, systemic-structural, environmental, and social factors.

Lack of awareness is limiting the usage of patient services which was mentioned by both groups. In recent years, significant advances in the mobile phone industry have led to a significant change in communication ways. Sending a short text message from health centres to the older adults has not been efficient, though it is not possible to call all of them. The older people can be informed through mass media (television and radio) in the current situation. Educated and healthy older adults usually have better information about innovative electronics and are more aware of health [17, 30]. This is a critical disconnect as older adults primarily rely on healthcare professionals to provide information about services [31].

Limited literacy of the older people and inattention to health issues may lead to non-seriousness in checking and treating diseases in the early stages. They usually refer to the hospitals over the terrible stage, resulting in higher costs for the older people and the community [32].

According to Bidarpoor and colleagues, the priority of treatment in the community is a reason for not referring from the perspective of service providers [17]. Safari and colleagues revealed that social and economic variables such as income and education might play a role in not referring. The majority of older adults do not work and have fewer options for continued income. They are at risk for rising costs of living including physician fee, transportation, medications, and paramedical procedures [33]. Moreover, economic status and income level also affect the number of older people patients referred to the centres. Those who cannot afford medications and laboratory tests may be reluctant to refer. However, those with good financial levels prefer to go to private centres instead of public ones, or they purchase medications and do tests without a doctor's order [30, 34–37]. A further study in Ghana indicated that despite the existence of a national health insurance scheme, health care services are not affordable and accessible for all people. Therefore, the use of services is delayed, or people are entirely excluded from health services [38]. Tajvar and colleagues also detailed that the high cost of health services is supposed as the main barrier. Not having a private car has not had a significant effect on reducing the older people to receive outpatient services [39].

Participants of the present study stated that the quality and quantity of equipment are not acceptable in most centres. The issue needs investigation to provide standard facilities and fund by the government and health policymakers. Furhtermore, Irani and colleagues realized that the type of disease, costs, and quality of services are associated with referring to healthcare centres [40]. Khayatan and colleagues also reported that according to employees of urban health centres, the quality of services provided by the centres was a significant factor in people's access to services [41]. The behaviour and performance of care workers also have a significant impact on the satisfaction and willingness of patients to return. The employees' competence, respect, and good treatment are the main satisfaction factors [42]. Unfortunately, some general practitioners and health workers have insufficient expertise and communication skills which contribute to not referring to and dissatisfaction of the older people.

Long-distance and transportation are other barriers to the older people's referral. The Integrated Aging Program was supposed to cover the people closest to the place of residence, but unfortunately, due to the limited capacity of the centres, renting the site, and the frequent relocation of the centres some patients have to travel a long way which is unpleasant and difficult for frail older people [30, 32, 33, 40, 43]. Moreover, the lack of social support (especially by children) and has led to the non-referral of some older peoples.

Insurance-related issues also affect people's satisfaction and referral to health centres. Many older peoples are worried about the cost of medications and lab tests due to the inefficiency of insurance and lack of services covered by the insurer, so they refuse to refer [31, 35, 39, 44].

The cleanliness and tidiness of the space and equipment are pleasant and essential for every person. This seems to be more critical for the older people. The presence of high-quality, hygienic toilet facilities, and suitable chairs in the waiting room affect the older people's satisfaction and referral. The unfavourable environment of the centres was shown to be a reason for not referring the cases. This is in line with Mohammad and colleagues, who stated that untidiness, inadequate facilities, and nasty buildings are the main reasons for dissatisfaction [42].

In the present study, general practitioners and primary healthcare providers stated that physical difficulties of the older adults were the main reasons which affecting other factors such as the unfavourable environment of the centres, access conditions, and transportation system. From point of care providers, further Iranian studies indicated that physical status was one of the influential factors [17, 32]. Older adults also felt that the services were counted useless. This might motivate people to refer to private centres. In line with this finding, a study showed that provision of inadequate services, people's indifference to receiving health facilities, and prioritizing to refer to the private sector were among the influential reasons for non-referral [17].

Client information and registration system deficiencies have been a problem that existed since the beginning of the health system and, unfortunately, continues. Low speed, frequent errors in completing information, frequent system disconnections, complicated and repetitive tasks for health care providers and general practitioners which is the shortcomings that, unfortunately, has not been fixed after several years. Clients' delays, dissatisfaction, and mental pressure occur following the health registration system [17].

## Conclusions

Both groups agreed on many aspects, including lack of education of the patients and lack of proper medical services. Existing problems in health care relate to both medical and non-medical factors. Improvement in health care delivery requires a deliberate focus on the patients’ specific needs. The restructuring of primary care provision was affirmed to have enhanced health provision for older people through increased efficiency, coordination, and quality. Due to the qualitative nature of this study, it is suggested to conduct quantitative and mixed studies in this field in the future. Carrying out targeted interventions to improve the quality of equipment in health care service centres, using health volunteers to identify and refer disabled and frail older people for training and receiving services, and holding educational sessions for care providers in the field of aging and how to deal with the older people it is suggested. A particular strength of this study was the inclusion of two different perspectives (older adults, general practitioners, and primary health providers). Possible limitations include the lack of gender insight in the findings.

## Supplementary Information

**Additional file 1: Table 3.** Why do older people not use the public health services of the integrated aging program? Reasons, Barriers and challenges (Group of older people: *n*=29). **Table 4.** Why do older people not use the public health services of the integrated aging program? Reasons, Barriers and challenges (Group of health workers: *n*=18).

## Data Availability

All data generated or analyzed during this study are included in this published article.
