# Supplementary material for: Why do older people not use the public health services of the integrated aging program? A multidimensional approach in a qualitative study
Source: BMC Health Serv Res. 2022 Oct 25;22:1288. doi: 10.1186/s12913-022-08689-6 (PMC9596180; doi:10.1186/s12913-022-08689-6)
Supplement: Supplementary file 1 — Additional file 1: Table 3. Why do older people not use the public health services of the integrated aging program? Reasons, Barriers and challenges (Group of older people: n=29). Table 4. Why do older people not use the public health services of the integrated aging program? Reasons, Barriers and challenges (Group of health workers: n=18). [file 12913_2022_8689_MOESM1_ESM.docx]

**Table 3- Why do older people not use the public health services of the integrated aging program? Reasons, Barriers and challenges (Group of older people: n=29)**

| **Theme** | **Subtheme** | **Statements** |
| --- | --- | --- |
| **Individual factors** | Following up only in the case of getting sick, self-medication, the idea of services uselessness, the preference or need to see a specialist, lack of knowledge about services, physical weakness, and having general practitioners in the family. | *“…Usually, if I get sick, I refer to a centre. It is not like they ask me to refer, for example, every three months. They do not call, so I do not refer them unless needed. They do not care about the patient, whether you are okay or are not...”*.  *“…For example, sometimes I have a heartbeat. Since my wife suffers from it, I take the same pills that they give her...”*.  *“…They do nothing, but I go to get a prescription and take medication…”*.  *“…When a specialist takes $2 for 5 minutes, the quality of her/his services is different. If s/he takes 15 cents, all of us will go inside...”*.  “… *I'm sick, and I have to go to my doctor. I cannot see any general practitioner, so I go to a specialist more often...”*.  *“…We do not have diabetes or other diseases; we do not deal much with family doctors...”.*  *“…My children are doctors. I do not need to refer to the centres. My daughter and brother are physicians, and his wife and nephew are pharmacists. If I need to see a doctor, I will refer a specialist. I do not need to go to a general practitioner...”.* |
| **Systemic-structural issues** | Lack of informing and calling for services, referral to other organizations or another therapist, absence of general practitioners, restrictions on prescribing, administering or supplying drugs, limited availability and affordability of mental health care services, lack of nutrition counselling and injection services, low-quality equipment, E-prescribing errors, lack of necessary doctor's expertise, the ineffectiveness of medical insurance, the small effect of doctor's prescription on the cost of drugs and tests, financial incapacity, and inefficiency of the referral system. | *“…We referred and registered for the family doctor, but I don't even know his/her name, or where s/he is. I have never visited him/her...”*.  *“…The family doctor messaged but did not call. They did nothing. Once, they messaged and asked if I was satisfied with the services or not. What should I say? I haven't referred, and you haven't done anything...”*.  *“…We are from the armed force. The organization that provides medical services to us do not know our information. We just go to the centre and ask for registration…”.*  *“…I referred several times, but the family doctor wasn't there. One of the doctors was my neighbour. I do not name him because he was also my mother's relative. They said the doctor was not here at the time, so go and come back two hours later. A family doctor is nonsense! I go to see if s/he is there or not! That is why I don't refer to the centre anymore...”*.  *“…To tell the truth, I do not see the family doctor unless to renew the medicine. S/he can't do much. S/he is allowed to write only a few medicines. If I get ill, I don't refer to a family doctor…”.*  *“…It will be good if a nutritionist and a psychologist are there. There is no psychologist to talk with if someone has a problem or the centre lacks nutrition services...”.*  *“…Their equipment is not first class. For example, the blood pressure device is not accurate. The care provider begins to test blood pressure and opens/closes it six times…”.*  *“…It's a big problem. We used to get medicine with a health insurance card quickly. Now they say we don't have access to the Internet; there is connection trouble, the prescription isn't readable, go back to the doctor and ask for what s/has written...”.*  *“…We have to say I feel hurt here or there, s/he prescribes acetaminophen, the diagnosis is not good. Or, I must say my blood sugar is high, then s/he tells me to take this or that medicine...”.*  *“…Testing is expensive. The insurer doesn't pay for the costs even with the stamp of a family doctor. The health insurance card is useless and now they do not accept the insurance card at all…”*.  *“…The health insurance card is something nonsense and useless. It is written, but we have to buy and pay which come out of our pockets. The insurer only gets the cash. It is in the interest of the insurance firm...”*.  *“…Due to busyness, sometimes I have to refer to the centre or the doctor later. The cost of medications is high, so we do not go much...”*.  *“…I referred to the specialist for my Lumbar Herniated Disc. He did not register in the system at all, so I didn't have a code to get medicine. He said because I do not have a contract with insurance, I do not write anything in the prescription. We have to pay for everything, so the insurance is useless...”*. |
| **Environmental aspects** | Lack of space for cars, lack of health and public facilities, and unfavourable environment of the health centres. | *“…The family doctor is in the city centre. There is no parking space. We usually have to walk…”.*  *“…There is no toilet. It is only for doctors, not for patients. You cannot go there when you need it…”.*  *“…The family doctor has a small office with rotten stairs and a steep slope because the building is old...”.* |
| **Social factors** | Fear of COVID-19 pandemic, lack of social support, and inability to establish interpersonal communication by health workers. | *“…Since the coronavirus has come, I haven't referred to the health centre or doctor...”*.  *“…These days, children do not feel like their parents. I do not leave anything to children in any way...*”.  *“…If you visit specialists in the office, they are concerned about all the details because they get the money and you have to come over again, but in the public centres, they behave you like an animal. They don't explain much... “*.  *“…The previous family doctor kept me waiting. He did not allow me to speak at all. He procrastinated me. He was impatient. He just fought. I finally changed him, and I was relieved…”*. |

**Table 4- Why do older people not use the public health services of the integrated aging program? Reasons, Barriers and challenges (Group of health workers: n=18)**

| **Theme** | **Subtheme** | **Statements** |
| --- | --- | --- |
| **Individual factors** | ‘Older people not taking the disease seriously’, preference or necessity of referring to a specialist, lack of knowledge about services, self-medication, physical and mental difficulties, forgetting the appointment, having a doctor in the family, and the idea of services uselessness | *“…The elderly does not take it very seriously. This needs to be addressed. They refer again only, for example, to get insulin…”*.  *“…Unfortunately, some of the elderly do not believe in seeing a general practitioner at all, and they like to just refer to a specialist...”*.  *“…For heart disease, everyone refers to a specialist. Some people call and say we just refer to our doctor…”*.  *“…Some people do not come because of physical incapacity. They rest just in bed and don't have the physical ability to refer to the centre…”.*  *“…Since I've retired and no longer have a position in other offices, my relatives have left me alone. I'm shocked and depressed. One of the cases said that if I were not afraid of God, I would have committed suicide...”*.  “…*Besides the pandemic that has reduced referrals, they also have memory impairment and so forget appointments. We call them as much as we can, though some are missed…*”.  *“…There are some elderlies who no longer need to refer to the centre because they have a doctor in the family. For example, there was one case whose son was a psychiatrist doing all the medical work for his parents. I called the elderly man, and he said, “I don’t need to refer to the centre…”*.  *“…Some elderlies say you do nothing there and I don't come! Their children believe the same and say we spend money on our elderly or their care. When we are far away, we have to hire a nurse. What can you do for us? Those with such a perspective cannot meet their needs with the centre, so they prefer not to refer…”*. |
| **Systemic-structural issues** | Lack of awareness, referring to private clinics, lack of rehabilitation services, no medicine provision, no follow-ups, inadequate equipment of centres, low-quality services due to lack of staff, low speed and flawed information registration system, shortcomings of electronic prescription and disruption of medicine reception, the COVID-19 pandemic, lack of expertise in the field of aging, the inefficiency of the referral system, inefficiency of insurance, and high costs of medical procedures. | *“…Many of older adults do not know who their family doctor is, where he works, how they can access the doctor, and what services the centre provides. They think we just provide vaccination and renew the medications. This is a drawback of the system...”*.  *“…There are so many clinics and specialists in the city that I've not seen them at all...”*. *“…Those with the insurance card of the Social Security Organization refer to the hospitals of the organization to reduce the costs…”*. *“…Much can be done in a public environment to encourage the elderly; for example, physiotherapy...”*.  *“…There are no special follow up programs. We have pressure control, height and weight check, but without medicine…” (A primary healthcare provider)*.  *“…In the city, the referral is optional, but care providers go to homes and follow up in villages. We do not have this possibility in the city...”*.  *“…We don't have a glucometer kit to check the patient's sugar, so we have to ask for or send a test. Those elderly who does not have the device can’t have cared for…”*.  *“…Only blood pressure is being screened here. We do not have a device for diabetes. Centres in the village have a glucometer, but those in cities don't. The rest of the screenings are done by phone, for example, colon cancer or fit testing… “*.  *“.. There is a lack of workforce in many centres. An expert may do three or four tasks for the elderly, middle-aged patients, pregnant women, and children. His/her workload is high, so the quality gets lower...”*. *“…The Internet is terrible. It gets disconnected repeatedly. I record on the system, and then I notice no connection. I have to do it over. This makes me tired, takes up all my time, and makes the patient wait behind the line...”*  *“…E-prescription gives error. We have to do it over all the time...”*.  *“…Those who need care don't refer to the centre due to the mass vaccination…”*.  *“…The capacity of the centres should be increased so that the elderly does not have to be covered by centres away from their home...”*.  *“…They should hold training courses for caring for the elderly. If they don't want to hire a person with the required expertise, at least they can provide the staff with training on treating the elderly...”*.  “…*About 50% of the prescriptions referred to a specialist are rejected. For example, they say the insurance does not pay them or does not pay on time. This makes the elderly complain and make them dissatisfied with us...”*.  *“…The medical costs and testing are so high that we can't afford. So, people don't refer to laboratories...”*.  *“…Higher ranked doctors often do not have a contract. We had a contract with an ophthalmologist, but he cancelled. The patient refers to the centre, and we don't have the doctor...!”*. |
| **Environmental aspects** | Lack of space for cars, lack of health and public facilities, and unfavourable environment of the health centres. | *“…Our centre is located in the worst spot. There is no place to park. Because most of older people are disabled, they have to park; the person who comes with old clients has to park the car and take the old patient to the centre. Nevertheless, often there is no parking space. It's a crowded place. If the elderly can, they will come themselves, but they need family support. So, the family may not accompany them, and the number of visits gets lower...”*.  *“…Unfortunately, we do not have a toilet for the client in the centre. It will be much better if the centre gets facilitated with toilet...”*.  *“…There is only one toilet usually used by the staff. The elderly may use it only in emergencies. No water cooler, no heating and cooling device. Though, since the corridor is small, I leave the door of my room open to cool down there...”.*  *“…Two doctors are in the same room. The patients complain about the lack of privacy. Why two doctors in the same room? They even vaccinate here. It's noisy...”.* And *“…Since some cases come from a long distance, our centre is not convenient for them. There is no taxi road, and they have to walk a bit. It is difficult to get to the centre…”.* |
| **Social factors** | Financial problems, fear of Covid 19, lack of social support | *“…Some of the cases do not refer, some refer rarely. They say we bought the medications. I ask why? They say you do corona test here. We are afraid...” (A general practitioner). “…There are cases that I call their children and s/he says you do not call me anymore, it does not matter to me, s/he stays with someone else, why don't you call him/his and I asked for the number of that person, but the child says you do not call me anymore. S/he even does not give the number of the person who cares the parent...”* *(A primary health provider)*.  *“…There is a travel fee. They have to come by taxi. That is expensive, so they do not come and wait for their child or someone else with a car to bring them to the centre...”* *(A primary health provider)*. |
